# 

**Published:** 1914-08-29

**Authors:** 


### CONTENTS.

| | PAGE |
|---|---|
| **The Heart of the British People... ... ...** | 585 |
| **Temporary Hospital Provision in War-Time ..** | 586 |
| **Hospital and Institutional News ... ... ...** | 587 |

Our Educational Number—An English Base Hospital for France—Hospital Secretaries on Active Service—Public Pharmacists at the Front—Infirmary Superintendents and Schemes of Public Work—The War's Effect on Panel Practice—A Corp of Masseuses for Active Service—The Queen's Naval Hospital, Southend—Convalescents and Home Districts—A Monastery as a Hospital—Some Panel Doctors' Grievances—Official Attitude to Auxiliary Home Hospitals—School Buildings as Military Hospitals—The Welsh War Hospital Scheme—The Transformation of Cambridge—Complaints Against a Wandsworth Infirmary—The Naming of Poor-Law Infirmary Wards—The Privacy of Sanatoria Patients—The Burnley Dispute—The Unoccupied Wards at Worcester Infirmary—The Combined War Scheme at Bristol—Institutional Pharmacists and Army Rank—Fifty Years of Hospital Dispensing—Poor-Law Infirmaries and their Drug Contractors—A General Drug Store for London—"Heather Day" in Manxland—A Corps for Cholera Service—The War and the Price of Drugs.

[*See over.*]

### CONTENTS—(*continued*).

PAGE

A STATE OF WAR.

**The Country and the Care of the Sick and Wounded** ... ... ... ... ... ... ... 593
Organisation and Control.

**Hospitals and their Contracts** ... ... ... ... 594

What Should Our Policy Be?

**Minor Hospital War News** ... ... ... ... ... 594

**The Position of Voluntary Hospitals** ... ... 595
The Drain on the Hospitals' Special Departments.

**Hospitals and the War** ... ... ... ... ... 596
The London Hospitals' Arrangements: St. Bartholomew's Hospital—West Ham and Eastern General Hospital—The London.
Provincial Hospital Arrangements: Aylesbury—Barnsley—Chatham District—Coventry and Warwickshire Hospital—Croydon — Doncaster and District—Felixstowe—Gloucester—Gravesend: An Emergency Test—Lancaster—Leamington—Salisbury Infirmary—St. Leonards, Hastings—Wakefield and District—Wolverhampton.
Poor-Law Infirmaries and the War Office: Birkenhead—Offers of Poor-Law Institutions—South Shields—An East Coast Town's Preparations.
Preparations in Scotland: Aberdeen, Glasgow, and Perth—Kilmarnock—Stirling—The Scottish Red Cross Society.
The Red Cross Society: The Red Cross Expedition to Belgium—The Red Cross Motor-car Corps—Progress of the Arrangements for Stores—The War Office and Private Offers—A Link Between Firing Line and Hospital—How Offers of Help are Dealt With—A Lady-Resident Volunteers—Red Cross Work at Cambridge—The Hospital Unit at Greenwich for Receiving Wounded in the Thames.

**The Editor's Letter-Box** ... ... ... ... ... 605

**A Noted Worker at Oldham** ... ... ... ... 606

**The Legal Position in Maternity Cases** ... 607
The Law, the Nurse, and the Compromise.

**Round the World** ... ... ... ... ... ... 608

Fellow-Workers and their Doings.

**The Institutional Worker** ... (Suppl.) 1
Hot Meals for Night Nurses.

